# Development and Field Evaluation of a Microbially-Inoculated Feather–Straw–Lignite Compost Material for the Reclamation of Post-Mining Soils

**DOI:** 10.3390/ma19143035

**Published:** 2026-07-14

**Authors:** Anna Choińska-Pulit, Justyna Sobolczyk-Bednarek, Dominika Kufka, Amelia Zielińska

**Affiliations:** “Poltegor-Institute” Opencast Mining Institute, Parkowa 25, 51-616 Wroclaw, Poland; anna.choinska-pulit@igo.wroc.pl (A.C.-P.); dominika.kufka@igo.wroc.pl (D.K.); amelia.zielinska@igo.wroc.pl (A.Z.)

**Keywords:** compost, *Bacillus*, *Streptomyces*, post-mining areas, feather, lignite

## Abstract

This study addresses degraded post-mining soil reclamation by developing a novel, microbially-inoculated waste-derived feather–straw–lignite organic-mineral compost material. Formulated from chicken feathers (20%), wheat straw (60%), and lignite (20%) to optimize the initial C:N ratio, the substrate was inoculated with a multi-strain complex (MIX) of *Bacillus altitudinis* 33, *Bacillus methylotrophicus* Alk, and *Streptomyces fulvissimus* K59. Testing progressed from laboratory scale to a 200 dm^3^ dynamic reactor and a 2025 field evaluation with maize (*Zea mays* L.) on sandy mining soils in Konin (Poland). Inoculation accelerated maturation, yielding a favorable C:N ratio of 12.09 and stabilized NH4-N of 0.03%. Peak dehydrogenase activity reached 16 DU by day 3. Field application enhanced sandy soil properties, increasing urease activity to 3.91 UU and providing 3.4 g/kg P_2_O_5_. Consequently, maize showed a highly significant 15% increase in average shoot height (30.23 cm vs. 25.88 cm in control; t(645) = 6.63, *p* < 0.001) and intense green coloration. However, absolute soil enzymatic activity remained low due to early spring moisture limitations and temperature constraints typical of temperate climates. These initial findings suggest that the microbially-enriched compost shows strong potential as a safe functional soil-reclamation material and compliant alternative to synthetic fertilizers.

## 1. Introduction

New solutions for post-mining land reclamation, revegetation, and ecosystem rehabilitation are currently receiving significant financial and regulatory support from the European Union. Initiatives aimed at improving soil health, such as the EU Soil Mission, the European Green Deal, and the Farm to Fork strategy (EU), have catalyzed the growth of the bioeconomy and regenerative agriculture. These trends are a direct response to global challenges, including soil degradation, climate change, and the urgent need to reduce mineral fertilizer consumption. Recent data indicates that the global organic fertilizer market is projected to reach approximately USD 13.6 billion by 2029, with some forecasts suggesting a value of nearly USD 20 billion by 2030 [1,2]. This robust growth, particularly in the United States and the European Union, reflects a fundamental shift in consumer preferences and regulatory frameworks away from synthetic inputs toward sustainable, ecological solutions.

Surface mining activities lead to diverse structural changes in both the landscape and topography [3]. A specific type of environmental degradation occurs when exhausted excavations are left empty, reach depths of several meters and expose various geological layers, such as sand, gravel, and clay [4]. Achieving successful and enduring restoration of former mining sites necessitates a cross-disciplinary strategy. This ensures a comprehensive plan for reviving the ecological, hydrological, and aesthetic values of the terrain. The chosen reclamation technique—whether biological, technical, or agricultural—is largely dictated by the physical characteristics of the land, soil quality, and the socio-economic framework [5]. The application of organic soil improvers like compost and sewage sludge for the restoration of impaired soils serves as a primary instance of biological remediation techniques.

Commonly used synthetic mineral fertilizers often lead to the gradual degradation of soil organic matter, negatively affecting its physical, chemical, and biological properties. In contrast, organic soil improvers enhance soil structure, water retention, and nutrient cycling, thereby contributing to long-term productivity and resilience. By promoting microbial activity and supporting the re-establishment of diverse plant life, organic amendments contribute to natural capital regeneration—a core objective of the circular economy. Transforming organic waste materials, such as feathers and straw, into valuable compost enables their reuse in the remediation of degraded soils while simultaneously conserving natural resources and energy [6,7,8]. Recent studies have demonstrated that waste-derived materials can serve not only as organic amendments but also as nutrient recovery agents. For example, Bouzar and Mamindy-Pajany [9] demonstrated that biomass bottom ash effectively removes and immobilizes phosphorus from both real and synthetic wastewater through calcium-phosphate precipitation mechanisms, highlighting the potential of industrial by-products within circular nutrient management systems. Their findings support the broader concept of transforming waste streams into value-added products for environmental restoration and sustainable resource recovery.

A significant global waste management challenge is posed by the poultry industry. Feathers account for approximately 5–10% of a chicken’s total mass [10], resulting in several million tons of this byproduct generated annually [11,12]. Improper management of this oversupply leads to severe environmental consequences, including long-term pollution and serious public health risks [13]. Traditionally, feathers have been processed via energy-intensive thermal treatment or high-pressure autoclaving to produce feed supplements with limited nutritional utility. Alternative disposal methods, such as incineration or landfilling, are equally problematic due to the release of nitrogen oxides, sulfur oxides, dioxins, and the contamination of soil and water [14,15].

The primary component of feathers is keratin, a fibrous structural protein characterized by a dense molecular network of hydrogen bonding, disulfide bridges, and extensive cross-linking [16]. While indigenous microflora possess some degradative potential, the natural process is often too protracted and inefficient [17]. Therefore, enriching the compost with specialized microbial inocula featuring keratinolytic activities is essential to optimize the biochemical transformation of feathers and straw [18]. Furthermore for recalcitrant protein-based wastes, decomposition can be accelerated through specialized rotary reactors or static piles with forced aeration, with optimal results [19].

In composts supplemented with external carbon sources, the inoculation of bacteria capable of biosolubilization is particularly critical. Biosolubilization involves the microbial liquefaction of coal, such as low-rank coal (LRC), which significantly enhances carbon bioavailability. This method facilitates the synthesis of complex organic substances, including humic and fulvic acids, which function as effective soil conditioners [8,20].

The composting process requires the monitoring of several key parameters, including physicochemical factors (pH), microbial activity (measured via enzymatic assays), and chemical indicators of maturity, such as the C/N ratio and the content of specific nitrogenous compounds. The C_org_/N_T_ ratio serves as a primary benchmark for compost maturity, with optimal initial values typically ranging from 20 to 30 [21,22]. To ensure high plant productivity, nitrogen should ideally be present in the form of nitrates (NO_3_), which are the most bioavailable species for plant uptake. Furthermore, the nitrification potential—evidenced by a decreasing NH_4_/NO_3_ ratio—acts as a definitive marker of maturation [23]. Beyond nitrogen dynamics, the availability of sulfur-bearing organic compounds is essential for preventing yield loss and maintaining the nutritional quality of crops [24]. Plant growth tests (often referred to as phytotoxicity tests or field tests) are the final, definitive step in evaluating the maturity and quality of compost. While chemical indicators provide data on stability, plant tests confirm whether the compost is actually beneficial for crop development [6]. Ultimately, composting process produces a stable, nutrient-dense product that may improve soil properties and supports the rehabilitation of lands degraded by mining activities.

The aim of this research was to design and evaluate a novel, microbially-inoculated organic-mineral composite material (engineered compost) tailored for the reclamation and functional restoration of degraded post-mining soils in the Konin region (Poland). To guide the investigation, three specific research hypotheses were proposed:

1. Inoculation of the waste mixture with a specialized multi-strain bacterial consortium (MIX) accelerates organic matter transformation, yielding a mature compost with a stable C:N ratio (<12).
2. Field application of this microbially-enriched compost to sandy post-mining soils significantly enhances soil biochemical quality by stimulating dehydrogenase, urease, and phosphatase activities.
3. The incorporation of the optimized compost improves nutrient availability, thereby significantly enhancing the vegetative growth performance and vigor of maize (*Zea mays* L.).

## 2. Materials and Methods

### 2.1. Substrates

The white broiler feathers were obtained from a local poultry-processing plant near Wrocław, Poland. The wheat straw came from a farm in Lower Silesia. The lignite dust was sourced from the Konin opencast mine, Poland.

### 2.2. Compost Inoculants

The composted material was inoculated with 3 selected strains belonging to the collection of the “Poltegor-Institute” in Wrocław:

1. ***Bacillus altitudinis* 33**—a proteolytic strain capable of synthesizing highly efficient keratinolytic enzymes responsible for the biochemical degradation of feather keratin.
2. ***Bacillus methylotrophicus* Alk**—a cellulolytic strain characterized by the capacity to synthesize cellulases, enabling the enzymatic hydrolysis of lignocellulosic plant biomass (e.g., wheat straw).
3. ***Streptomyces fulvissimus* K59**—a specialized actinobacterium exhibiting strong biosolubilization activity toward low-rank coal (lignite dust), facilitating the conversion of recalcitrant carbon into bioavailable organic compounds.

### 2.3. Reclaimed Area

**Figure 1:**
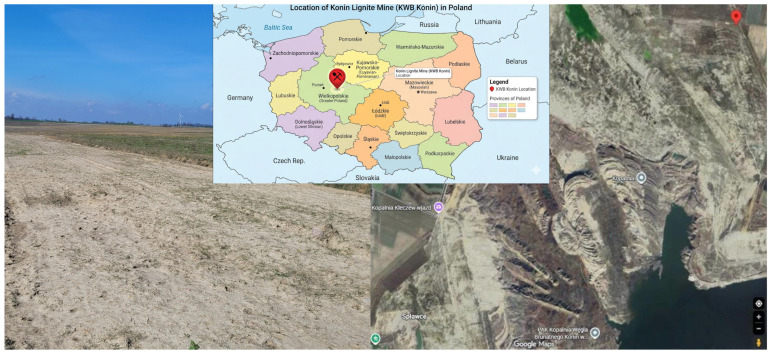
Photographic documentation of the plots with their exact locations.

The reclamation area was located within the Konin open-pit mine in Kleczew, Poland. The sampling site was located at the following coordinates: 52.430956° N, 18.174571° E. Soil samples were collected for physicochemical, chemical, biochemical, and microbiological analyses to establish the baseline characteristics of the site prior to reclamation. A plot of land located on a degraded mining site belonging to the Konin mine was selected for field testing. A photograph of the plot section, including its precise location, is presented in Figure 1. The experimental area was divided into three sections: a test plot, a control plot, and an isolation strip situated between the two. The initial phase of the research involved the collection and preliminary analysis of soil samples intended for remediation. These samples were evaluated for their physicochemical and chemical properties. Additional information related to the location is included in the publication Maksymowicz and Musiałek [25].

### 2.4. Feather Degradation in Solid-State Cultures and Compost

The first stage of solid-state cultures (laboratory compost) was carried out 41 days in 18 laboratory containers odorless Mini Garland Compost Bin with a capacity of 7 L, made of plastic and equipped with a carbon filter and two semi-technical compost containers (dynamic with capacity of 200 dm^3^). Laboratory (200 g) and semi-technical batches (60 kg) were prepared from raw feathers (cut to the length of 3–4 cm), straw (particle size 3–4 cm), and lignite. Straw, feathers, and lignite were mixed at a weight ratio of 60:20:20 (*w*/*w*) to achieve an initial Corg/Nt ratio of 20. Each component exhibits a distinct functionality within the composite matrix: wheat straw serves as a porous structural skeleton that ensures mechanical aeration; chicken feathers act as a densely cross-linked, keratinous organic depot for the sustained release of nitrogen; and lignite functions as a mineral sorbent and humic substance precursor, stabilizing the material’s microstructure.

The liquid compost inoculum consisted of three microbial strains: *Bacillus altitudinis*, *Bacillus methylotrophicus*, and *Streptomyces fulvissimus*. Each strain was cultivated individually in a sterile liquid Yeast Peptone Glucose (YPG) medium (BTL sp. z o.o., Łódź, Poland). Cultivations were performed in an orbital shaker at 180 rpm and a constant temperature of 30 °C. The incubation period was 24 h for both *B. altitudinis* and *B. methylotrophicus*, and 72 h for *S. fulvissimus*. To ensure the required cell density (OD_600_), the growth of each strain was monitored. The final viable cell concentration of each strain was determined using Koch’s pour plate method and adjusted to 1 × 10^8^ CFU/mL based on a previously established growth curve. Following incubation, the individual cultures were combined in equal volume ratios (1:1:1, *v*/*v*/*v*) to form the final multi-strain compost inoculum (MIX). In this final mixture, the total bacterial cell density was maintained at 1 × 10^8^ CFU/mL, with each strain constituting exactly one-third of the population. Consequently, the application of this inoculum at a 10% (*w*/*w*) dose provided an initial total bacterial load of 1 × 10^7^ CFU/g of the compost mass. The inoculum was applied once at the beginning of the composting process. Uninoculated mixture containing exclusively autochthonous microflora served as a control.

The next stage of the research involved composting, which was carried out for 41 days in 200 dm^3^ dynamic reactor system equipped with a horizontal mixer at an ambient temperature of 20–25 °C. Dynamic reactor system included a compressor unit, air supply lines, frequency converters, a control box, and electrical and control cables. The system was also equipped with forced ventilation using an HL 275/50 compressor (Airpress Polska Sp. z o.o., Przeźmierowo, Poland) (30 m^3^ per day). The compost biomass was turned periodically for 5 min, 20 times a day.

During solid-state cultures and composting, temperature moisture content were monitored. The compost moisture content was adjusted to 60% every 7 days using tap water. Samples were collected on days 0, 3, 10, 17, 35, and 41 of the process. The collected samples of solid-state cultures (laboratory scale) and compost (semi-technical scale) were mixed into a homogenous mixture, which was then used for enzymatic, physicochemical, and chemical analyses.

### 2.5. Field Tests

The field experiment was conducted during the 2025 growing season on a reclaimed post-mining area at the Jóźwin IIB site in Konin, Poland (52.430956° N, 18.174571° E). The experimental area comprised three singular, large-scale treatment zones (82 m^2^ each): (i) an unamended control plot (sown, without compost), (ii) an untreated and unsown isolation strip (7.5 m width) acting as a buffer zone to prevent cross-contamination, and (iii) the experimental plot treated with the inoculated compost. The experimental design utilized singular large-scale treatment zones where the heights of individual plants were systematically measured.

Preliminary physicochemical analyses of samples collected across the site verified highly homogeneous sandy substrate conditions, with similar texture and chemical composition throughout the study area. In November 2024, the microbially-inoculated compost was applied to the treated plot at a fixed rate of 150 kg per plot, which is equivalent to approximately 1.83 kg/m^2^ (or 18.3 t/ha).

In April 2025, both the control plot and the compost-treated plot were sown with maize (*Zea mays* L.; Hodowla Roślin Smolice Sp. z o. o. Grupa IHAR, Kobulin, Poland). Each plot was divided into nine rows, with each row sown with 48 seeds (totaling 432 seeds per plot). Sowing was carried out in a strict grid pattern with a spacing of 0.75 m between rows and 0.25 m between plants within a row. No auxiliary mineral fertilizers or artificial irrigation were applied during the trial.

To account for any residual spatial heterogeneity within the post-mining site, a spatial sampling strategy was employed. Soil samples were collected post-harvest using a soil core sampler from the topsoil layer (15–30 cm) at 9 predefined random points distributed in a zigzag pattern along each plot. The collection scheme was included in the article by Maksymowicz and Musiałek [25]. The collected soil cores were thoroughly mixed, quartered, and divided into independent composite replicates for subsequent chemical, physicochemical, and biochemical analyses. For biometric plant monitoring, individual plant shoot lengths were recorded at maturity across all rows. Data were analyzed using Welch’s *t*-test for independent samples (α = 0.05) to evaluate the significance of plant growth enhancement.

### 2.6. Physiochemical Analysis

Moisture content in the soil and compost samples was determined gravimetrically after drying at 105 °C. The pH of the soil and compost was measured by suspending a 2 g sample in 20 mL of distilled water (or tap water, if specifically required), stirring for 30 min, and then performing the measurement [26]. The temperature of the composted material was recorded daily based on five measurements taken at random locations at a depth of 50 cm. All physicochemical analyses were performed in triplicate.

### 2.7. EDX

The preliminary determination of the soil chemical composition involved conducting a semi-quantitative analysis using an energy-dispersive X-ray fluorescence (EDXRF) spectrometer (EDX-7000, Shimadzu, Kyoto, Japan). Prior to measurement, the soil samples underwent initial preparation, including air-drying at ambient temperature, impact crushing, powdering using an IKA M 20 universal mill. Then, to obtain the desired grain size fraction (<100 µm), the samples were manually sieved through a standard laboratory sieve with a nominal mesh size of 0.1 mm. The quantities of the components, i.e., powdered rock material (2.25 ± 0.1 g) and a binding agent (0.75 ± 0.1 g) in the form of boric acid (CHEMPUR, Piekary Śląskie, Poland) were weighed. The components were mechanically mixed and homogenized for approximately one minute in a porcelain mortar to ensure sample homogeneity and achieve uniform distribution of the binder throughout the sample volume. The mixture was then placed in the manual die of a Specac Atlas™ Manual hydraulic 15 Ton, by Specac, Orpington, UK. The pressing process was carried out under a pressure of 15 tons for 3 min, resulting in discs with sufficient mechanical strength and structural uniformity, which is necessary for precise EDXRF analysis. The prepared discs were then analysed using an energy-dispersive X-ray fluorescence spectrometer (EDX-7000, Shimadzu, Kyoto, Japan). Based on the results, the composition of the compost mixture (C:N:S ratio) was formulated.

### 2.8. Determination of Dehydrogenase Activity in Solid-State Cultures

Dehydrogenase activity was determined with a modified method of the Casida [27] method for 2,3,5-triphenyl tetrazolium chloride (TTC) (Pol-Aura, Dywity, Poland) and expressed in μmol of 1,3,5-triphenylformazan (TPF) (Pol-Aura, Dywity, Poland) formed within 20 h per 1 g of compost d.m. (dehydrogenase unit—DU; μmol TPF·g^−1^ dm·20 h^−1^). All analyses were performed in triplicate.

### 2.9. Determination of Phosphatase Activity

Acid phosphatase activity in the soil was determined according to the method by Tabatabai and Bremner [28]. At the beginning, 1 g of fresh, moist soil (sieved through a 2 mm mesh) was placed into three separate flasks. To establish the required environment, a Modified Universal Buffer (MUB) at pH 6.5 was used, composed of 1 M NaOH, Tris (hydroxymethyl) aminomethane, maleic acid, boric acid, and citric acid monohydrate (CHEMPUR, Piekary Śląskie, Poland). Two flasks served as technical replicates, while the third was used as a control. To each of the two sample flasks, 1 mL of p-nitrophenyl phosphate (PNP) solution (CHEMPUR, Piekary Śląskie, Poland) (10 mg/mL) and 4 mL of MUB (pH 6.5) were added. The control flask received only 4 mL of MUB initially. All flasks were swirled and incubated at 37 °C for 1 h. Following incubation, 1 mL of the PNP substrate was added to the control flask to account for non-enzymatic substrate interference. To terminate the reaction and extract the released p-nitrophenol, 1 mL of 0.5 M CaCl_2_ and 4 mL of 0.5 M NaOH were added to all flasks, followed by 90 mL of distilled water. The soil suspensions were then filtered. The absorbance of the resulting filtrates was measured at λ = 400 nm using a VWR UV-Vis spectrophotometer (VWR International BVBA, Leuven, China). Measurements were performed against a blank solution (95 mL distilled water, 1 mL 0.5 M CaCl_2_, and 4 mL 0.5 M NaOH). The final enzyme activity (PU) was expressed as µg of p-nitrophenol per gram of dry soil per hour (µg p-NP·g^−1^ dm·h^−1^). All analyses were performed in triplicate.

### 2.10. Determination of Urease Activity

Soil urease activity (UU) was assessed following the protocol developed by Tabatabai and Bremner [29]. The procedure relied on measuring a colored complex formed with phenylnitroprusside and sodium hypochlorite (CHEMPUR, Piekary Śląskie, Poland). Freshly collected 1-g soil samples were placed in conical flasks, treated with a pH 6.7 citrate buffer and a 10% urea solution (CHEMPUR, Piekary Śląskie, Poland), and subsequently incubated at 37 °C for 1.5 h. Following incubation, the mixture was extracted using 2M KCl. After filtration and the addition of coloring reagents, absorbance was measured at 630 nm utilizing a VWR UV-Vis spectrophotometer. The resulting enzyme activity was defined as the amount of µgN-NH_4_^+^·g^−1^ soil dm·2 h^−1^. All analyses were performed in triplicate.

### 2.11. Chemical Analysis of Compost and Soil

Mineral and fertilizer analyses of selected compost and soil samples and their individual components were carried out at the Institute of Geography and Regional Development, University of Wrocław. Prior to measurement, the soil samples underwent initial preparation, including air-drying at ambient temperature, impact crushing, powdering using an IKA M 20 universal mill. Then, to obtain the desired grain size fraction (<100 µm), the samples were manually sieved through a standard laboratory sieve with a nominal mesh size of 0.1 mm. The study included determinations of individual fractions of carbon, nitrogen, and sulfur, with organic carbon (C_org_) measured by the oxidimetric method using K_2_Cr_2_O_7_. Ammonium and nitrate nitrogen (N-NH_4_ and N-NO_3_) concentration were measured spectrophotometrically in water extracts 1:100. Total sulfur (S_T_) was determined in the CS-MAT 5500 instrument (Strohlein GmbH & Co., Kaarst, Germany, currently Bruker AXS Inc., Madison, WI, USA). The total contents of selected macroelements (P, K, Mg, Na, Ca) and microelements (Ni, Fe, Cr, Pb, Zn, Mn, Cu, Cd) were determined by the ICP (Inductively Coupled Plasma) method (ICP-OES Thermo Scientific iCAP 7400, Waltham, MA, USA) after mineralization (Microwave Digestion System—Start D, Milestone Helping Chemists, Shelton, CT, USA) with a mixture of concentrated hydrochloric and nitric acid in a volume ratio of 3:1. The total Hg content was measured on an AMA analyzer (MA-2, Nippon Instruments Corporation, Kyoto, Japan). The chemical analyses were performed in duplicates (*n* = 2); this setup was justified by the thorough mechanical homogenization, milling, and sieving processes applied to the raw matrices before analysis, which substantially minimized sampling error.

### 2.12. Statistical Analysis

To compare the mean plant height between the study group (*n* = 337) and the control group (*n* = 331), Welch’s *t*-test for independent samples was used. This test was chosen because of the lack of homogeneity of variance in the compared groups and the different sample sizes. The homogeneity of variance was verified by F-Snedecor test (*p*-value < 0.00012). Calculations were performed at a significance level of α = 0.05. Analysis was performed using the Analysis ToolPak in Microsoft Excel (Microsoft Office LTSC Professinal Plus 2024).

Inferential statistical testing (Welch’s *t*-test) was exclusively restricted to the plant biometric data due to the large population of independent biological observations (n1 = 337, n2 = 331). Physicochemical properties, trace elements, and soil enzymatic activities were evaluated using composite pooled samples from each large-scale treatment zone, meaning that their laboratory replicates represent analytical precision rather than independent ecological variance, precluding the valid use of comparative hypothesis testing.

## 3. Results and Discussion

### 3.1. Preliminary Soil Tests

The results indicated that the soil was sandy, with a low moisture content (9.16%) (Table 1). Sandy soils typically exhibit the lowest fertility due to their limited water-holding capacity, high permeability, and low nutrient concentration. However, the application of compost can enhance water retention; for instance, Dolit et al. [30] demonstrated that food waste compost yielded a significant increase (169%) in readily available water, raised total porosity by 6%, and reduced bulk density by 64%. This interaction not only improves water and nutrient sequestration in sandy soils but also promotes plant growth and overall vigor.

### 3.2. Laboratory-Scale Composting

The first stage of composting was conducted on a laboratory scale to select the most effective inoculum. Simultaneously, a compost mixture with a specific C:N ratio was developed. To ensure optimal compost maturity, it is essential to maintain an initial C_org_/N_T_ ratio between 20 and 30—a threshold widely recognized in the literature [21,22]. To determine the final composition and proportions, an elemental analysis of the individual components was performed (Table 2). The ternary formulation consisting of 20% feathers, 60% wheat straw, and 20% lignite was determined as optimal through an optimization mass-balance calculation based on the elemental data from Table 2 and our previous findings regarding keratin waste composting [8]. This specific configuration was engineered to achieve an initial C/N ratio of 20.7 (Table 5) while preventing excessive ammonia emission and to guarantee sufficient matrix porosity and structural aeration in the dynamic reactor system. This mixture of feathers, straw, and lignite was then composted using various inocula: indigenous microflora (control), three single-strain inocula: *Bacillus methylotrophicus* Alk (cellulolytic) [31], *Streptomyces fulvissimus* K59 (lignite biosolubilization) [32], and *Bacillus altitudinis* 33 (proteolytic) and a multi-organism complex inoculum (MIX = 33 + Alk + 59).

**Figure 2:**
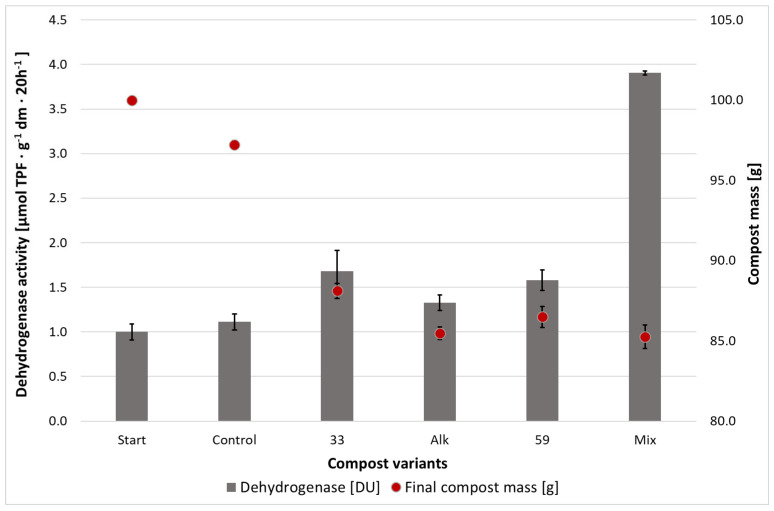
Dehydrogenase activity and final dry mass of laboratory composts. Error bars indicate mean ± SD.

During the process, dehydrogenase activity and dry matter loss were monitored (Figure 2). Dehydrogenases are intracellular enzymes found exclusively in the living cells of soil microorganisms, such as bacteria and fungi, and they catalyze oxidation–reduction reactions during cellular respiration. These enzymes are considered an excellent indicator of microbial biological activity because they reflect the intensity of microbial metabolic processes. Furthermore, they are directly involved in the decomposition of organic matter, which releases nutrients that subsequently become available to plants [33]. Dehydrogenase activity was selected as the foundational screening parameter at the laboratory scale due to its capacity to reflect the global, intracellular respiratory flux of viable biomass. Unlike extracellular hydrolases (such as cellulases or keratinases), which are prone to severe non-specific adsorption and immobilization onto the highly porous matrices of lignite and wheat straw—thereby distorting extraction yields—intracellular dehydrogenases provide a highly reliable, real-time index of microbial energetic adaptation and survival on the targeted mixed substrate. The highest composting efficiency, indicated by both dry matter loss and dehydrogenase activity, was observed with the combined MIX inoculum. As demonstrated in Figure 2, the individual monocultures achieved significantly lower total dry mass loss and lower peak dehydrogenase activity compared to the combined MIX formulation. The maximum composting efficiency was strictly locked to the MIX consortium, proving that the combined metabolic pathways of proteolytic, cellulolytic, and coal-solubilizing strains may operate synergistically, where the primary degradation products of one strain serve as metabolic substrates for the next. Research conducted by Li et al. [34] investigated the integration of chicken feathers into both chemical (5% NaOH) and biological (*Chryseobacterium indologenes*) pretreatment protocols for chicken manure composting. Their findings indicate that the biological pretreatment of chicken feathers was the most effective relative to the control group. A similarly beneficial effect of compost inoculation was observed by Sarkar et al. [35]. The authors reported that inoculating vegetable waste compost with thermophilic *Geobacillus* sp. enhanced dehydrogenase activity and lowered the C:N ratio, thereby confirming the effectiveness of the inoculum. Furthermore, the superior effectiveness of multi-strain inocula compared to monocultures in the composting process was corroborated by Wang et al. [36]. This study evaluated the performance of single and composite *Bacillus* inoculants (*Bacillus methylotrophicus* F-6, *Bacillus velezensis* T-B, and the F-6/T-B complex) in enhancing polylactic acid (PLA) degradation during the composting of cattle manure and wheat straw. The results demonstrated that the multi-strain inoculant (F-6/T-B) exhibited the most pronounced pro-degradation effect.

### 3.3. Semi-Technical Scale Composting

**Figure 3:**
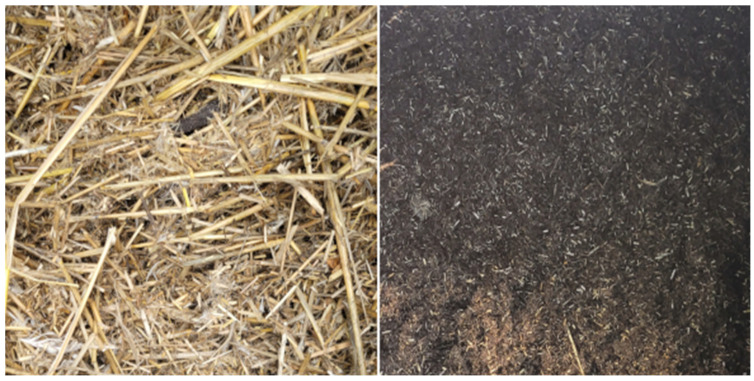
Macrostructural transformation of the ternary compost formulation before (day 0, **left**) and after the composting process (day 41, **right**), showing the transition from raw components to a homogeneous, soil-like humus.

After the completion of the laboratory-scale composting phase, the next step was a semi technical-scale test (60 kg of compost). The selected compost mixture, enriched with a multi-strain inoculum, was used in both dynamic composters (200 dm^3^), where the composting process was monitored. A uniform particle size is critical for both easy spreading and proper soil aeration. A mature, stable compost should have a pleasant, earthy smell, while foul or rotten odors indicate ongoing anaerobic decomposition and the presence of undesirable organic acids [37]. The product from the dynamic composting process fully met these criteria (Figure 3).

**Figure 4:**
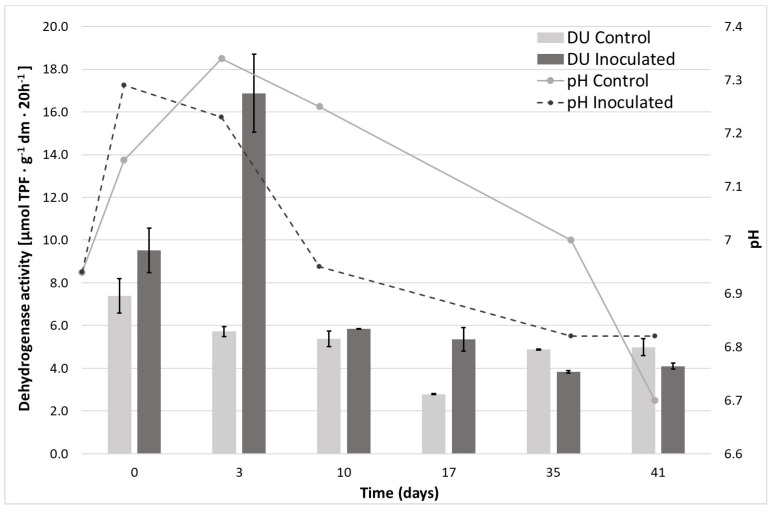
Dehydrogenase activity and pH in the control and inoculated compost. Error bars indicate mean ± SD.

Throughout the composting process, key parameters such as pH and microbiological activity indicated by dehydrogenase activity were monitored. During composting, the pH of both the control and experimental composts remained similar, ranging from 6.7 to 7.4 (Figure 4). A slight alkalization of the compost mass was observed within the first 10 days and may have been related to the progressive decomposition of protein (feather keratin). Notably, the inoculated compost reached alkaline pH levels more rapidly—as early as the third day—which was likely influenced by the inoculation with the keratinolytic bacterium *Bacillus altitudinis* 33. According to research by Korniłłowicz-Kowalska [38], in addition to proteolytic and keratinolytic activity, the alkalization of the culture medium—associated with the release of soluble proteins and amino groups—serves as a key indicator of the keratinolytic potential of microorganisms.

Research by Mondini et al. [39] suggests that enzymatic activity, due to its relationship with organic matter dynamics, can function as a criterion for assessing compost maturity and the overall progression of biodegradation. A significant increase in microbial activity, expressed as dehydrogenase enzyme activity, was observed in the inoculated compost on the third day of the process (16 DU) (Figure 4). Slightly lower dehydrogenase activity values (11 DU) were reported by Sobolczyk-Bednarek et al. [8] for feathers composted in a static reactor system. This discrepancy may be attributed to the superior aeration of the compost mass in the rotary reactor compared to the static system, as dehydrogenase activity levels directly reflect the metabolic activity of the microflora. It must be noted that while dehydrogenase activity serves as a proxy for global microbial metabolism, it reflects the collective respiration of both the inoculated strains and the indigenous microflora. The rapid spike to 16 DU on day 3 in the inoculated setup—absent in the control—indicates that the introduced MIX strains successfully initiated the metabolic surge. Subsequently, the microbial breakdown of recalcitrant keratin and cellulose matrices released low-molecular-weight nutrients, which likely exerted a priming effect, stimulating the metabolic activity of the native microflora and contributing to the sustained elevated dehydrogenase activity throughout the process.

During the composting process, organic compounds are transformed into minerals that can be used to fertilize the soil, which is the basis of recycling. In the tested composts, a decrease in organic carbon content was observed with increasing composting time. The most intensive transformation of organic carbon (C_org_) occurred in the inoculated compost (Table 3). The total nitrogen content (N_T_) and its individual forms in the studied composts increased with the duration of the process. The highest nitrogen increase was recorded in the inoculated compost. The main source of nitrogen in the compost mass was the keratin proteins of feathers, which were biodegradable, while nitrogen from the amino groups of amino acids was released through deamination. Some of this nitrogen was lost through release into the environment, but the majority of it underwent nitrification and denitrification. Bacteria release ammonium nitrogen mainly in the high-temperature stage of organic decomposition. As pH levels rise, this nitrogen transforms into ammonia gas, leading to a substantial loss of total nitrogen in the final compost [37]. Consequently, enriching the nitrogen levels post-composting is a strategic way to enhance the value and effectiveness of compost for agricultural applications [40,41]. This element plays a significant role in determining the fertilizing value of compost. Release of soluble nitrogen fractions in the compost mirrors the degradation of the insoluble protein matrix (keratin) into highly bioavailable soluble forms. In turn process of mineralization and loss of organic matter led to an increase in the total sulfur content in the compost, from initial value 0.4% to 0.53% in control and 0.58% in inoculated compost.

The compost material was analyzed to determine the content of essential fertilizing components, including nitrogen, phosphorus, potassium, and organic matter (Table 4). Nutrient Content (NPK): The concentrations of macronutrients like total nitrogen (N_T_), phosphorus (P), and potassium (K) determine a compost’s direct fertilizer value. While compost is generally a slow-release fertilizer, a lab analysis provides a precise nutrient breakdown. The progressive mineralization process occurring in the compost caused an increase in the macronutrient content in the environment. In the tested composts, as the experiment progressed, the content of these nutrients increased by 3% (K_2_O) to 62% (P_2_O_5_) compared to the initial mixture. In the control compost, changes in the content of individual macronutrients occurred more slowly than in the inoculated test compost after the same time. Pursuant to the Polish Regulation of the Minister of Agriculture and Rural Development of 9 August 2024, on the implementation of selected provisions of the Act on Fertilizers and Fertilization [42], solid organic fertilizers must contain at least 30% organic matter on a dry matter basis. Furthermore, if nitrogen, phosphorus, or potassium (or their sum) are declared, the content of these components must not be less than: 0.3% (*m*/*m*) total nitrogen (N_T_), 0.2% (*m*/*m*) phosphorus expressed as phosphorus pentoxide (P_2_O_5_), and 0.2% (*m*/*m*) potassium expressed as potassium oxide (K_2_O). The compost obtained in this study meets the criteria set forth in this regulation regarding the content of C_org_, N_T_, K_2_O, and P_2_O_5_.

Based on the results of the tests carried out so far on individual mineral components, the final assessment of the compost was made, determining their chemical maturity indexes (Table 5). The basic factor determining the proper course of the composting process is the appropriate carbon to nitrogen ratio in the starting material, which should be in the C/N range of 20–30. A high C:N ratio (greater than 25:1) indicates that the compost is not fully decomposed, which can lead to nitrogen immobilization, making it unavailable to plants. In mature compost, this ratio should be lower, C/N < 12 [43]. Only inoculated compost met this criterion, as the C/N index = 12. A comparable C/N ratio of 15 was reported by Chand et al. [44], who, consistent with the methodology of this study, composted feathers using a bacterial inoculum. These findings align with the research by Li et al. [34], which demonstrated that biological pretreatment of chicken feathers is the superior approach for accelerating organic matter breakdown and enhancing humus development.

From the perspective of the fertilizing value of compost, an important maturity index is the nitrogen ratio N-NH_4_/N-NO_3_, which reflects the degree of oxidation of mineral nitrogen. When the N-NH_4_/N-NO_3_ ratio equals one, it is assumed that the processes related to nitrogen transformation have begun to stabilize. In the starting material, the NH_4_/NO_3_ ratio was 0.02, while the test and control composts achieved the same result of NH_4_/NO_3_ = 0.01. However, a high NH_4_/N ratio was observed in the initial sample indicating incomplete decomposition of organic matter in the compost. The inoculated and the control compost achieved the desired value of this ratio (<0.04%). The C/N ratio and nitrogen mineralization dynamics serve as key indicators of the biochemical and thermodynamic stability of the developed material, ensuring it does not undergo uncontrolled degradation upon environmental deployment. Raw lignite is inherently resistant to attack by indigenous microorganisms; however, the presence of *Streptomyces fulvissimus* K59 in the MIX inoculum, a strain with previously confirmed [32] abilities, is highly desirable. This strain should contribute to the biosolubilization of this fine-grained coal fraction, which likely contributed to the final product achieving a highly mature, stabilized C:N ratio of 12.09 (Table 5).

The full biochemical maturity and structural stability of the microbially-inoculated compost are robustly supported by the convergence of multiple independent indicators. While the final C/N ratio reached an optimized baseline of 12.09 (Table 5), its safety profile is corroborated by an advanced state of nitrification, evidenced by the NH_4_/NO_3_ ratio stabilizing at 0.01 and the reduction of free ammonium nitrogen to a non-hazardous level of 0.03%. Ultimate functional maturity was validated in vivo through the seasonal field cultivation trials described in Section 3.4.

Table 6 presents the concentrations of selected trace elements determined by ICP–OES analysis in compost samples collected at the initial and final stages of the process, including the mature control compost. The analyzed elements included chromium (Cr), cadmium (Cd), copper (Cu), nickel (Ni), lead (Pb), mercury (Hg), cobalt (Co), and zinc (Zn). The assessment of heavy metal concentrations in organic amendments is essential due to potential environmental risks, as long-term application and accumulation of trace elements in soil may negatively affect soil fertility, microbial activity, crop quality, and contribute to groundwater contamination through leaching and surface runoff [45].

Within the European Union, permissible concentrations of heavy metals in fertilizing products are regulated by Regulation (EU) 2019/1009 [46]. In Poland, the requirements for solid organic fertilizers are additionally specified in the Regulation of the Minister of Agriculture and Rural Development of 9 August 2024 [42], which establishes the following maximum permissible limits (mg kg^−1^ DM): Cr—100, Cd—5, Ni—60, Pb—140, and Hg—2.

Based on the obtained results, cadmium and cobalt concentrations remained below the limit of detection (LOD) throughout the composting process, indicating negligible contamination of the raw materials with these toxic elements. Similarly, mercury concentrations remained very low, reaching a maximum of 0.028 mg Hg kg^−1^ DM in the final control compost, which represents less than 2% of the national statutory threshold.

Chromium concentrations increased during composting, from 19 mg Cr kg^−1^ DM in the initial material to 51 and 53 mg Cr kg^−1^ DM in the final inoculated and control products, respectively. This increase was related to the progressive mineralization of organic matter and the concentration effect commonly observed during compost maturation, resulting from mass reduction and organic carbon losses during biodegradation [47,48]. Despite this trend, the final chromium concentration reached approximately 51–53% of the national threshold. Nickel concentrations ranged from 10 to 25 mg Ni kg^−1^ DM, remaining more than two-fold lower than the permissible limit (reaching up to 42% of the threshold). Lead contents also remained low, reaching a maximum of 10 mg Pb kg^−1^ DM in the mature matrices, which is approximately fourteen times lower (7%) than the legal limit.

Copper and zinc concentrations were evaluated against the limits defined by Regulation (EU) 2019/1009 [46]. The copper concentration in the final composts reached 7–9 mg Cu kg^−1^ DM, fulfilling the requirements and remaining more than thirty-fold lower than the European limit. Zinc concentrations ranged from 24 to 28 mg Zn kg^−1^ DM, which was also approximately thirty times lower than the maximum permissible European threshold.

Overall, the obtained results confirmed that the produced organic-mineral compost met all applicable Polish and European regulatory standards, rendering it environmentally safe and suitable for agricultural and land reclamation applications.

However, because only total trace element concentrations were determined to evaluate statutory compliance, no direct conclusions can be drawn regarding their long-term environmental behavior, mobility, or bioavailability following soil application. In this context, the behavior of lignite-associated trace elements merits specific attention. Although lignite may naturally introduce trace amounts of metals, it is characterized by a high content of humic substances. These macromolecules possess numerous reactive functional groups (e.g., carboxyl and phenolic groups) capable of reducing the availability of trace elements through adsorption, ion exchange, complexation, and the formation of stable organo-mineral associations [49,50,51].

### 3.4. Field Testing of Compost Influence on Soil Parameters and Plant Growth

Soil fertility is a set of physical, chemical, and biological properties that provide suitable conditions for plant growth. Among these components, biological factors are crucial, including the enzymatic activity of the soil environment. Soil enzymatic activity is a highly sensitive indicator of soil properties. The particular importance of enzymatic activity for the functioning of soil microorganisms makes these indicators widely used to determine soil biological activity. The main groups of enzymes studied in soil are oxidoreductases (dehydrogenases) phosphatases and ureases. The high biochemical activity of these enzymes in the soil means they play a crucial role in shaping the ecological stability and productivity of agroecosystems and are perceived as a good indicator of soil quality [49].

The microbiological activity of the tested soil, expressed as dehydrogenase activity, was highest in the soil treated with compost (Table 7). A comparable phenomenon was reported by Wyszkowska and Wyszkowski [50], where the application of compost in soil contaminated with petroleum, positively influenced the activity of various soil enzymes, including dehydrogenase. However, the value of this parameter in present study was relatively low, as the activity of this group of enzymes should ideally reach values of several units. This is likely due to low soil moisture and the season (spring, autumn), when the activity of soil microflora is still relatively low.

Phosphorus is a key macronutrient for plants, essential for root growth, flowering, and fruiting. In soils poor in available phosphorus, phosphatase activity is an important mechanism increasing the bioavailability of this element. These enzymes catalyse the hydrolysis of organic phosphorus compounds (e.g., phytate and phospholipids) in the soil. Their main function is to convert phosphorus from unavailable to available forms. There are two main types of phosphatases: acidic (active in soils with a pH < 6.5) and alkaline (active in soils with a pH > 7.0) [49]. Their activity depends on soil pH, allowing them to function effectively across a range of soil conditions. Similar to dehydrogenases, high phosphatase activity indicates good biological soil health and its ability to provide nutrients to plants. A deficiency of available phosphorus in the soil stimulates both plants and microorganisms to increase phosphatase production. Furthermore, as observed in this study, phosphatase activity was higher in spring, when temperatures rise, compared to results obtained in November or March, when ambient temperatures often drop below 10 °C and night frosts occur (Table 7). Moreover, higher activity of this enzyme was observed in the compost-amended soil compared to the control soil. Despite similar phosphatase activity across treatments, the inoculated compost exhibited significantly higher inorganic phosphorus (3.4 g/kg P_2_O_5_), likely due to the inoculated microflora’s metabolic action. This aligns with findings by Ortega-Torres et al. [51], who demonstrated that *Pseudomonas aeruginosa* and specialized enzymatic cocktails can enhance phosphorus mineralization in mature compost, increasing P release by up to 95%. The observed increase in available phosphorus is consistent with broader research on phosphorus mobilization and recovery from waste-derived materials. Bouzar and Mamindy-Pajany [9] reported that biomass bottom ash enhanced phosphorus retention through calcium-mediated precipitation and hydroxyapatite formation. Although the mechanism identified in the present study is primarily biological and associated with microbial mineralization of organic phosphorus compounds, both approaches demonstrate the potential of waste-based materials to improve phosphorus cycling and nutrient availability in environmental applications.

Soil urease is a key catalyst in the hydrolysis of urea into ammonia, which is then oxidized by nitrifying bacteria into nitrates. This biochemical pathway is essential for enhancing the efficiency of nitrogen uptake by plants [49]. Studies have shown that the optimal temperature for urease activity in soil is often in the range of 60–70 °C, which is significantly higher than typical soil temperatures. However, higher temperatures in summer and early autumn significantly accelerate urea hydrolysis. Urease requires water to carry out the chemical reaction, and its activity decreases during dry periods. Kumari and Rao [52] investigated the impact of temperature on soil urease activity across four distinct soil types. Their findings revealed that enzyme activity—measured as the amount of NH_4_^+^ released per gram of soil per hour—increased significantly with temperature, rising from 0.9 µg NH_4_^+^·g^−1^·h^−1^ at 20 °C to 2.16 µg NH_4_^+^·g^−1^·h^−1^ at 30 °C. These observations suggest that the ambient temperatures typically recorded in Poland during March and April (below 20 °C) are not conducive to high urease activity in the examined soil. Summer and early autumn often provide an optimal combination of high temperature and adequate soil moisture (e.g., after rainfall), which promotes maximum enzyme activity. In the tested soil samples, lower enzymatic activity was observed in April compared to samples collected in November and March of the previous year, which may be due to seasonal changes in environmental conditions and the activity of soil microorganisms. However, urease activity (Table 7) in the soil amended with compost (3.91 IU) was higher than in the control soil (2.86 IU), indicating a stimulating effect of organic matter on soil biological activity. These results are consistent with literature reports [53,54] indicating that organic amendments increase urease activity, while the use of urease inhibitors along with nitrogen fertilizers can temporarily reduce it. These observations are consistent with previous findings presented by Yang et al. [55].

**Figure 5:**
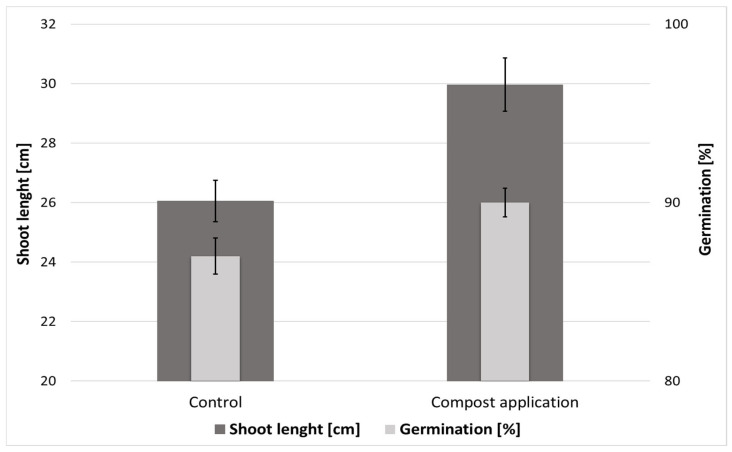
Average maize shoot length of the control and test plots (with compost application). Error bars indicate mean ± SD.

**Figure 6:**
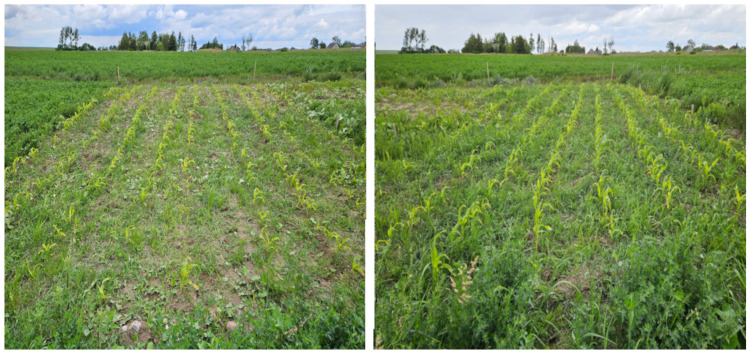
Field test: control without compost (on the **left**) and with compost application (on the **right**).

To assess the effect of compost on plant growth, a three-month field study was conducted. Shoot length was measured on maize plants grown in both a control plot and a compost-treated experimental plot. The data, collected from nine separate rows, revealed a statistically significant difference: plants cultivated with compost exhibited a 15% increase in height compared to the control group (Figure 5). Welch’s *t*-test analysis revealed statistically significant differences in plant height between the compost treated and control groups. Plants treated with compost reached, on average, a higher height (M = 30.23) compared to the control group (M = 25.88). This difference was highly statistically significant: t(645) = 6.63; *p*-value < 0.0001. These results represent a successful case study of multi-strain inoculum application in this specific post-mining location, which supports the conclusion that compost may be effective soil amendment for enhancing plant growth and can serve as a functional soil-reclamation material. The visual differences between the two groups are illustrated in Figure 6. The soil application of compost derived from chicken feathers (CFC) was also evaluated by Chand et al. [44]. Consistent with the present study, their compost was inoculated with *Bacillus* bacteria, which enhanced tomato growth relative to the control group. The researchers concluded that a 10% CFC concentration serves as an excellent organic fertilizer for tomato cultivation.

The plants growing in the plot with compost added had a more intense green color compared to the plants growing in the control plot (Figure 6). Joardar et al. [6] observed similar effect in his studies. Authors prepared compost by burying feathers in soil for 3 months and then drying. This resulted in the product-treated poultry feather waste (TPFW). They observed that plants treated with TPFW were greener than control plants, and the green color became more pronounced with increasing TPFW application intensity. The authors concluded that this was due to the fact that treated feathers can provide a good source of nitrogen for plants.

Unlike conventional synthetic fertilizers that provide immediate nutrient release but lack structural value, or raw organic wastes that degrade unpredictably, the developed functional compost material exhibits balanced material stability. The porous structure of the straw skeleton combined with the high exchange capacity of lignite creates a functional matrix that simultaneously improves soil water retention and serves as a carrier for targeted soil microorganisms. The material safety evaluation by emphasizing that the heavy metal concentrations (Hg, Cd, Pb, Cr, Ni) are well below the strict threshold limits man-dated by European Regulation (EU) 2019/1009 [46], proving the high eco-compatibility and safety performance of the compost material.

Applying this microbiologically enriched compost to permeable, sandy, post-mining soils can have significant short- and long-term ecological impacts. In the short term, successful remediation is evidenced by a rapid increase in soil enzyme activity (dehydrogenase, urease, and phosphatase) during the first growing season. This increase is directly correlated with a 15% increase in *Zea mays* L. shoot height and a transient increase in plant-available nutrients (P_2_O_5_, K_2_O). However, the high macroporosity and low cation exchange capacity (CEC) of sandy soils can lead to accelerated organic matter mineralization and potential nutrient leaching (NO_3_) during heavy rainfall. Therefore, long-term monitoring is indicated and should focus on: (i) the multi-year dynamics of soil organic carbon (Corg) accumulation and humification, (ii) the long-term stability of introduced *Bacillus* and *Streptomyces* populations after initial substrate depletion, and (iii) periodic assessments of soil water retention curves to verify whether the lignite–feather matrix provides lasting structural improvements or only temporary benefits.

### 3.5. Scalability, Techno-Economic Feasibility, and Regulatory Boundaries

Transitioning the feather–straw–lignite organic-mineral composting process from a 200 dm^3^ pilot bioreactor to an industrial-scale operation involves navigating several critical techno-economic, logistical, and regulatory boundaries. From a regulatory perspective, the primary bottleneck concerns product classification and biosecurity. Raw chicken feathers are legally classified as Category 3 Animal By-Products (ABPs) under Regulation (EC) No 1069/2009 [46]. Consequently, transforming this waste stream into a commercial fertilizing product requires strict adherence to validation parameters for processing plants. Logistical boundaries represent another critical factor governing scalability. Due to the high moisture content of raw poultry waste and the bulk density of wheat straw, long-distance transportation is economically non-viable and ecologically counterproductive due to the associated transport emissions. Therefore, full-scale deployment must adopt a regional “hub-and-spoke” model, where composting facilities are strategically localized within a 50–100 km radius of centralized poultry processing plants and agricultural hubs. The inclusion of lignite dust introduces geographic constraints; due to its bulk density, long-distance transportation is economically unviable. Therefore, production plants should ideally operate under a decentralized model, located in close proximity to both open-pit mines and intensive poultry farming hubs to minimize logistics costs. Economically, the formulation offers a highly competitive outlook. The primary ingredients—chicken feathers and wheat straw—represent zero- or negative-cost waste streams, where poultry processors often subsidize disposal costs, effectively turning raw materials into a revenue source (gate fees). These financial advantages substantially offset the operational expenditures (OPEX) related to forced aeration and inoculation with the specialized MIX culture. Given rising global synthetic fertilizer prices and the increasing penalties on industrial waste landfilling, this circular economy model provides a robust, self-sustaining financial framework capable of delivering a high-value, heavy-metal-compliant soil amendment at a fraction of the market cost of conventional mineral fertilizers. Furthermore under the current European Union Regulation (EU) 2019/1009 [46] and the Polish Regulation of the Minister of Agriculture and Rural Development (9 August 2024) [42], the final product fits the rigorous criteria for ‘Solid Organic Fertilizer’ or ‘Soil Improver’, thanks to its favourable nutrient content (N_T_, P_2_O_5_, K_2_O) and low heavy metal profile. However, because it contains a deliberate multi-strain bacterial inoculum, it must also navigate the regulatory frameworks governing biostimulants. Maintaining precise batch-to-batch microbial consistency is mandatory to secure commercial registration and compliance within the EU circular economy framework.

### 3.6. Limitations of the Study and Future Perspectives

While the developed microbially-enhanced ternary compost demonstrated robust agronomic performance and safety profiles, several inherent limitations of this study must be acknowledged. The field validation was restricted to a single-season pioneer vegetative monitoring cycle (the 2025 season) conducted at a localized experimental plot within the Konin post-mining area in Poland. Consequently, the long-term durability of the organic matter, multi-year crop yield dynamics, and the generalizability of these specific formulation kinetics to highly heterogeneous post-mining soils globally remain to be fully established. Due to the scale of the semi-technical operation, process monitoring relied on highly homogenized composite pooling, which limited the number of distinct replicates for certain physicochemical variables. Furthermore, while global metabolic activity was robustly tracked via dehydrogenase activity, direct molecular bio-tracking of individual inoculated populations (e.g., via qPCR or 16S rRNA sequencing) was not performed. Although the total heavy metal content fully satisfied domestic and European regulatory criteria, empirical leaching, surface runoff, and lysimeter tracking were not executed within this initial baseline phase. To fully address the long-term environmental fate and potential accumulation of these elements during systematic application, future research should incorporate complementary testing approaches. These should include CaCl_2_ and DTPA extractions to determine plant-available fractions, standardized leaching procedures (e.g., EN 12457 or TCLP), and sequential extraction methods to characterize trace element speciation and binding forms [56,57,58]. Furthermore the long-term environmental behavior of trace elements derived from lignite-amended composts remains insufficiently understood. In particular, changes in soil pH, organic matter decomposition, and redox conditions may influence metal speciation and mobility over time. Future research trajectories will focus on multi-year, multi-site monitoring grids incorporating high-throughput sequencing and targeted nutrient/metal leaching dynamics to comprehensively optimize this circular bioeconomy model for industrial ecological restoration.

## 4. Conclusions

A compost mixture was developed from three waste streams: chicken feathers (20%), wheat straw (60%), and lignite (20%), aiming to achieve an initial C:N ratio of 20–30. A multi-strain microbiological inoculum (MIX), consisting of *Bacillus methylotrophicus* Alk, *Streptomyces fulvissimus* K59, and *Bacillus altitudinis* 33, was selected for its superior enzymatic properties, including proteolytic and cellulolytic activities, as well as carbon biosolubilization. The multi-strain inoculum accelerated carbon transformation and promoted faster maturation compared to the uninoculated control. Composting on a semi-technical scale (60 kg) yielded high-quality compost characterized by an improved earthy structure, enhanced organic matter decomposition, and a favorable mature-phase C:N ratio (<12), meeting all key chemical maturity indicators. The obtained results confirmed that the produced compost fulfilled both Polish and European regulatory requirements regarding heavy metal concentrations in organic fertilizers. The optimized compost was subsequently applied to a post-mining experimental field in Konin, Poland. Its agronomic efficacy was assessed by monitoring maize (*Zea mays* L.) growth and yield throughout the 2025 growing season. Field experiments confirmed the effectiveness of the compost as a soil amendment; corn grown with the compost exhibited a statistically significant 15% increase in shoot height compared to the control group. Furthermore, the compost served as an excellent source of essential minerals while demonstrating elevated microbiological activity, as indicated by dehydrogenase levels. Ultimately, this microbially-enhanced compost acts as a functional soil-reclamation material and multi-component nutrient supplement that effectively improved selected short-term indicators of soil fertility and showed potential for post-mining soil reclamation. While the positive morphological response of *Zea mays* L. and the enhanced enzymatic activities are highly promising, it must be noted that these results are derived from a single-season trial on singular large-scale plots. Consequently, further multi-replicate plots and long-term monitoring are required to fully generalize the ecological efficacy of this material across heterogeneous post-mining landscapes.

## 5. Patents

Sobolczyk-Bednarek, J., Choińska-Pulit, A. Compost Inoculum, a Method for Producing Compost Inoculum, and a Method for Compost Biodegradation. Polish Patent Application P.455181 (WIPO ST 10/C PL455181), 23 March 2026. Applicant: “POLTEGOR-INSTYTUT” Instytut Górnictwa Odkrywkowego.

## Figures and Tables

**Table 1:** Chemical and physiochemical parameters of tested soils samples. Data values are presented as the mean ± SD.

Type of Analysis	Parameter	Mean Value
Physiochemical	pH	7.30
	moisture content [%]	9.16
		
Chemical analysis (EDX)	SiO_2_	72.15 ± 2.31
[wt. %]	Al_2_O_3_	11.57 ± 0.56
	CaO	6.38 ± 0.62
	Fe_2_O_3_	3.19 ± 0.27
	K_2_O	3.01 ± 0.05
	MgO	1.78 ± 0.24
	TiO_2_	0.78 ± 0.02
	SO_3_	0.46 ± 0.09
	P_2_O_5_	0.22 ± 0.09
	Na_2_O	0.21 ± 0.07
	ZrO_2_	0.11 ± 0.02
	MnO	0.07 ± 0.003
	Cr_2_O_3_	0.03 ± 0.003
	CuO	0.02 ± 0.001

**Table 2:** Elemental analysis of compost components.

Components	C	H	N	S
	[%]			
Lignite	48.38	4.19	0.62	1.54
Feathers	46.45	6.34	15.13	1.95
Wheat straw	43.70	5.24	0.38	0.01

**Table 3:** Contents of selected elements and different carbon fractions in compost.

Compost	N	H	S	C	C_organic_	C_inorganic_
				[%]		
Start	2.15	6.43	0.4	43.52	43.52	0.00
Control	2.93	6.24	0.53	42.96	42.96	0.00
Inoculated	3.50	5.97	0.58	42.29	42.29	0.00

**Table 4:** The content of available forms of selected elements and macroelements in compost.

Compost	SO_4_	PO_4_	NO_3_	NH_4_	K_2_O	MgO	P_2_O_5_
	mg/kg DM ^1^						
Start	4457.9	513.4	810.8	17.7	3620.3	1662.0	2108.3
Control	4147.1	379.7	739.7	5.38	3251.7	2066.2	1765.7
Inoculated	3698.2	756.9	941.1	9.27	3719.6	1840.8	3413.5

^1^ DM—Dry mass.

**Table 5:** Indicators of compost maturity.

Compost	C/N	NH_4_/NO_3_	NH_4_-N [%]
Start	20.7	0.02	0.08
Control	14.68	0.01	0.02
Inoculated	12.09	0.01	0.03

**Table 6:** Trace metal content in compost samples.

Compost	Hg	Cr	Ni	Pb	Cd	Cu	Co	Zn
mg/kg DM ^1^								
Start	<LOD ^2^	19	10	9	<1.25	7	<2.5	24
Control	0.028	53	25	10	<1.25	9	<2.5	28
Inoculated	<LOD ^2^	51	25	9	<1.25	7	<2.5	26

^1^ DM—Dry mass. ^2^ LOD—Limit of detection.

**Table 7:** Enzymatic activity in control soils and soils with compost application in various vegetation seasons. Data values are presented as the mean ± SD.

Date	Soil Sample	Dehydrogenases [DU]	Phosphatases [PU]	Urease [UU]
March 2024	Untreated	0.02 ± 0.001	65.27 ± 3.0	4.83 ± 0.3
November 2024	Untreated	0.01 ± 0.001	62.06 ± 4.2	9.21 ± 0.4
April 2025	Control-untreated	0.04 ± 0.005	83.39 ± 4.6	2.86 ± 0.0
April 2025	Compost	0.07 ± 0.009	89.43 ± 0.5	3.91 ± 0.2
August 2025	Control-untreated	0.04 ± 0.000	127.31 ± 2.2	0.55 ± 0.0
August 2025	Compost	0.09 ± 0.000	126.88 ± 0.5	0.55 ± 0.1

## Data Availability

The original contributions presented in this study are included in the article. Further inquires can be directed to the corresponding author.

## Abbreviations

The following abbreviations are used in this manuscript:

MIX	Multi-strain microbiological inoculum
LRC	Low rank coal
EDXRF	Energy-dispersive X-ray fluorescence
ICP	Inductively Coupled Plasma
TCA	Trichloroacetic acid
TTC	2,3,5-triphenyl tetrazolium chloride
PNP	p-nitrophenyl phosphate
